# Head Impact Exposure in Junior and Adult Australian Football Players

**DOI:** 10.1155/2018/8376030

**Published:** 2018-04-01

**Authors:** Mark Hecimovich, Doug King, Alasdair Dempsey, Myles Murphy

**Affiliations:** ^1^Division of Athletic Training, University of Northern Iowa, Cedar Falls, IA, USA; ^2^School of Psychology and Exercise Science, Murdoch University, Murdoch, WA, Australia; ^3^Sports Performance Research Institute New Zealand (SPRINZ) at AUT Millennium, Faculty of Health and Environmental Science, Auckland University of Technology, Auckland, New Zealand; ^4^School of Science and Technology, University of New England, Armidale, NSW, Australia; ^5^School of Physiotherapy, The University of Notre Dame Australia, Fremantle, WA, Australia; ^6^Medical Department, Peel Thunder Football Club, Mandurah, WA, Australia

## Abstract

This study measured and compared the frequency, magnitude, and distribution of head impacts sustained by junior and adult Australian football players, respectively, and between player positions over a season of games. Twelve junior and twelve adult players were tracked using a skin-mounted impact sensor. Head impact exposure, including frequency, magnitude, and location of impacts, was quantified using previously established methods. Over the collection period, there were no significant differences in the impact frequency between junior and adult players. However, there was a significant increase in the frequency of head impacts for midfielders in both grades once we accounted for player position. A comparable amount of head impacts in both junior and adult players has implications for Australian football regarding player safety and medical coverage as younger players sustained similar impact levels as adult players. The other implication of a higher impact profile within midfielders is that, by targeting education and prevention strategies, a decrease in the incidence of sports-related concussion may result.

## 1. Introduction

Australian football (AF) is a contact, invasion game [1] combining athleticism with speed, necessitating skillful foot and hand passing [2] with aggressive tackling and sudden collisions between players and the ground [1, 3]. These collisions have the potential to result in head impacts that may lead to sports-related concussion (SRC), a mild traumatic brain injury associated with a range of symptoms, including headache and impaired memory [4, 5].

The SRC incidence rate for adults in AF (games and training) has been documented at 0.5 (95% CI: 0.4 to 0.7) per 1,000 participation hours and, in games only, it is 1.2 (95% CI: 0.8 to 1.7) per 1,000 game hours [6]. In the 9- to 17-year-old group the overall incidence rate has been reported at 0.6 (95% CI: 0.2 to 1.0) per 1,000 Athlete Exposures (A-E) with the older group (ages 14–17) recording 0.8 (95% CI: 0.1 to 1.5) per 1,000 A-E [7].

A recent study [8] measured the frequency, magnitude, and distribution of head impacts in senior AF players that revealed an average of 213 impacts per player for the season resulting in 29 impacts per player per match. The quantification of head impacts in sport is documented in the literature using a variety of devices. For example, mouthguard or head-mounted sensors (XPatch; X2Biosystems, USA) have documented head impacts in rugby union [9, 10], rugby league [11], collegiate wrestling [12], and AF [8]. This data has enabled the development of analytical risk functions [13–16], concussion risk curves [14], and risk weighted exposure metrics [17], further assisting in the identification of athletes at risk of SRC.

Most studies examining head impacts have been on American football, rugby, and ice hockey at various levels [4, 9, 11, 18, 19]. Recently, in AF, the level of impacts in adults were established [8]. However, there are no reported levels in juniors and the differences in head impacts between playing positions are unknown.

In AF, there are 18 players on field, per team, during game activities, with up to four players utilized as interchange players [20]. The interchange players can be rotated onto the field as frequently as the coach requires. Players partake in one of three positional groups, forwards, midfielders, and defenders [2]. Forwards typically mark the ball (catch the ball on the full from a kick greater than 15 m) in the best position possible and then take a free kick at the goals; midfielders typically work alongside forwards and defenders, picking up loose balls from the ground or regaining possession and clearing the ball from their goal area; and defenders typically attempt to “spoil” the opposition forwards marking the ball [2]. The roles of the forwards are to mark the ball and this is done by jumping or jostling against defenders and midfielders in an attempt to get the ball. In doing this, defenders will be attempting to interfere with the forwards as they try to mark the ball and this can result in contact with the top of the head, knocking the player out of the way resulting in the forward and defender recording impacts to the top of the head or may fall onto the group resulting in the higher linear acceleration being recorded [8, 11]. If variations are observed between player positions, further research to identify what game-related activities result in these differences and, due to the free-flowing and complex nature of each position, more data are required on each player in order to determine the level of risk associated with the number of impacts to the head [8].

Research into the differences in head impacts between junior and senior playing levels is important as it informs sporting organizations on how much medical coverage is required. For example, if head impacts are small and infrequent in juniors when compared to seniors it makes sense that increased cover is required for senior competitions.

While the correlation between the measurements of head impacts and developing SRC is poor, it is well established that actual head impacts are a requirement of developing SRC [21]. Current strategies to improve player safety and reduce the incidence include providing education in preventing head impacts. Player safety may be improved through rule changes, behaviour changes, and better on-field identification of potentially injurious impacts. However, the biggest challenge is the identification of who is most at risk of developing SRC to best target education strategies. Specifically, AF research into the mechanics of head impacts may demonstrate differences in the incidence and magnitude of head impacts depending on age and playing position.

The aims of this study were as follows.

(1) Investigating the frequency, magnitude, and distribution of head impacts sustained by junior and senior players over a playing season

(2) Investigating the frequency, magnitude, and distribution of head impacts between player positions

## 2. Material and Methods

### 2.1. Participants and IRB

A prospective observational cohort study was conducted in junior and senior AF players during games over the course of the 2016 season. A total of 12 juniors (18 ± 0.7 yr.; 183.5 ± 6.6 cm and 79.1 ± 6.9 kg) and 12 adults (21 ± 2.2 yr.; 187.4 ± 7.3 cm; 81 ± 7.7 kg) players agreed to participate and were enrolled in the study. These players consisted of 8 forwards (5 juniors, 3 adults); 8 midfielders (3 juniors, 5 adults); and 8 defenders (4 juniors, 4 adults). The ruck position players (3 juniors) were imbedded with the forward position as they move into the forward line when not competing in ruck contests. Consent was obtained from the players before enrolling in the study. The researchers' university ethics committee approved all procedures (MUHREC 2016/012). The participating teams and players provided approval prior to commencing the study.

### 2.2. Impact Testing

Participants wore a skin-mounted impact sensor (XPatch, X2 Biosystems, USA) that was fitted behind the right ear over the mastoid process using adhesive patch, as per manufacturer's instructions, and secured with a clear skin dressing. This application and data extraction were piloted successfully during the 2015 season using Injury Management Software (IMS) (X2 Biosystems) [11].

The sensor contained a low-power, high-*g* triaxial accelerometer with 200*g* maximum per axis, and a triaxial angular rate gyroscope to capture six degrees of freedom for linear and rotational time history acceleration of the heads center of gravity for all impacts that occurred during games. The time history incorporated three axes (*x*,* y*,* z*) of acceleration and velocity. While upright these planes describe the medial-lateral, anterior-posterior, and vertical acceleration and deceleration. The IMS enabled the raw data to be transformed to the approximate head center of gravity by using a rigid-body transformation for linear acceleration and a 5-point stencil for rotational acceleration [9, 22]. The biomechanical measures of head impact severity consisted of impact duration (ms), linear acceleration (*g*), and rotational head acceleration (rad/s^2^). Resultant linear acceleration is the rate of change in velocity of the estimated center of gravity of the head attributable to an impact and the associated direction of motion of the head [23]. Resultant rotational acceleration is the rate of change in rotational velocity of the head attributable to an impact and its direction in a coordinate system with the origin at the estimated center of gravity of the head [23]. False impacts were removed by the X2Biosystems proprietary “de-clacking” algorithm [9]. Impacts with a resultant linear acceleration of <10*g* were removed. The remaining impacts were downloaded and time-filtered to include only those impacts that occurred during game participation.

Head impact exposure including frequency, magnitude, and location of impacts were quantified using previously established methods [24, 25]. The impact variables were not normally distributed (Kolmogorov-Smirnov; *p* < 0.001). Three measures of impact frequency were computed for each player:* player impacts*, the total, median, 25th–75th interquartile range (IQR), and the 95th percentile of head impacts recorded for a player during all the games observed,* player group impacts*, the total, median [IQR], and the 95th percentile of impacts recorded for each of the player groups (forwards, midfielders, and backs) during all games observed, and* impacts per game*, the total, median [IQR], and the 95th percentile of head impacts recorded for a player during all the games observed.

Player head impact exposures were assessed utilizing previously published levels for injury tolerance (linear > 95*g* and rotational acceleration > 5,500 rad/s^2^), impact severity (linear mild < 66*g*, moderate 66–106*g*, severe > 106*g*), and rotational acceleration (mild < 4,600 rad/s^2^, moderate 4,600–7,900 rad/s^2^, severe > 7,900 rad/s^2^) [26–31].

Two additional risk equations were included in the analysis of the head impact exposure data. The Head Impact Telemetry Severity profile (HIT_SP_) [32] is weighted composite score including linear and rotational acceleration, impact duration, and impact location. The Risk Weighted Exposure Combined Probability (RWE_CP_) [17] is a logistic regression equation and regression coefficient of injury risk prediction of an injury occurring based on previously published analytical risk functions. RWE_CP_ combines resultant linear and rotational acceleration to elucidate individual player and team-based head impact exposure. The HIT_SP_ and RWE_CP_ were analyzed by player position and player group impacts utilizing a Friedman repeated measures ANOVA on ranks. A post hoc analysis with Wilcoxon signed-rank tests was conducted with a Bonferroni correction applied if any significant differences were observed.

Resultant peak linear and rotational acceleration and impact locations (front, back, side, and top) between player positions were assessed utilizing a Friedman repeated measures ANOVA on ranks. A post hoc analysis with a Wilcoxon signed-rank tests was conducted with a Bonferroni correction applied if any significant differences were observed. A one sample chi-squared (*χ*^2^) test and risk ratio (RR), with 95% confidence intervals (CI), were utilized to determine whether the observed impact frequency was significantly different from the expected impact frequency. Statistical significance was set at *p* < 0.05.

## 3. Results

A total of 1,609 impacts were recorded over the duration of the study resulting in an average of 60 ± 36 impacts per player per season (Table 1). Peak linear acceleration ranged from 10*g* to 158.8*g* with a median and 95th percentile value of 15.3*g* and 45.8*g*, respectively. Rotational acceleration ranged from 52.3 rad/s^2^ to 22,458.0 rad/s^2^ with a median value of 2,729.8 rad/s^2^. As a result, the HIT_SP_ varied from 10.7 to 207.5 with a median value of 15.5. Junior players had a median resultant peak linear and rotational acceleration of 15.1*g* and 2,741.7 rad/s^2^. Senior players had a median resultant peak linear and rotational acceleration of 15.7*g* and 2,757.4 rad/s^2^.

Midfielders recorded more impacts than defenders for both the junior (RR: 1.6 [95% CI: 1.4–1.8]; *p* = 0.0017) and senior teams (RR: 1.3 [95% CI: 1.1–1.5]; *p* < 0.0001) (see Table 1). Junior midfielders recorded a higher median peak linear acceleration (15.5*g*) than defenders (*χ*^2^ = 34.0; *p* < 0.0001; *z* = −2.01; *p* = 0.0443). Senior forwards recorded a lower median peak linear acceleration (15.8*g*) than midfielders (*χ*^2^ = 28.9; *p* < 0.0001; *z* = −2.29; *p* = 0.0221). Senior midfielders recorded a higher median resultant peak linear acceleration (16.9*g*) than junior midfielders (15.5*g*; *χ*^2^ = 16.5; *p* < 0.0001; *z* = −2.03; *p* = 0.0421).

The side of the head was the most common impact site for total (37.4%) and senior (43.4%) players but the front of the head (37.9%) was the most common impact site for juniors (Table 2). Juniors recorded noticeably more impacts to the front of the head (RR: 1.2 [95% CI: 1.0 to 01.4]; *p* = 0.0366) and less impacts to the side of the head (RR: 1.4 [95% CI: 1.2–1.5]; *p* = 0.0002) than seniors. For total impacts the front of the head recorded noticeably higher resultant peak rotational acceleration (3,278.8 rad/s^2^) than the top of the head (2,310.3 rad/s^2^; *χ*^2^ = 14.2; *p* = 0.0002; *z* = −2.9; *p* = 0.0036). As a result, the front of the head recorded a higher HIT_SP_ (15.6 versus 14.6; *χ*^2^ = 4.5; *p* = 0.0339; *z* = −2.1; *p* = 0.0379) and RWE_CP_  (0.0012 versus 0.0005; *χ*^2^ = 15.5; *p* = 0.0004; *z* = −2.7; *p* = 0.0077) when compared with the top.

Less than 1% of peak resultant linear impacts greater than 95*g* for junior, senior, and total impacts were recorded (Table 3). The majority of peak resultant linear impacts were recorded in the mild (<66*g*) category for juniors (95.4%) and seniors (96.7%). The majority of peak resultant rotational acceleration was in the mild (<4,600 rad/s^2^) severity for juniors (73.2%) and seniors (71.0%). There were more severe HIT_SP_ impacts recorded for juniors (RR: 1.8 [95% CI: 1.0–3.2]; *p* = 0.0461) when compared with seniors.

## 4. Discussion

This study compared head impact mechanics at two levels of play and the differences in impacts between positions. It is important to stress that it remains unknown as to how many head impacts and what intensity impact might lead to SRC. The severity of brain injury among young athletes is also reported to be due to multifactorial components and not solely impact forces [23, 33].

This study adds to the current literature as it recorded the magnitude, frequency, and distribution of impacts to the head for different positions in AF players participating at two distinct levels: junior and senior. A recent study [8] established measures of frequency, magnitude, and distribution of head impacts in adult-level AF and this study furthers these findings by contrasting two age groups.

### 4.1. Differences between Senior and Junior Players

In this study, there were similar results between the two levels of play in median linear and rotational acceleration with the senior players recording slightly higher values in both areas. This is not surprising due to the increased speed, game duration, and players size in senior players [34, 35]. Although the age levels did not vary substantially and are representative of the cohorts, this remains problematic as the differences in measurements were negligible and potentially place the younger cohort at increased risk of SRC. The Kennard principle [36] states that a young brain is more adaptive and protective against damage than an adult brain; however, research suggests that the developing brain is more vulnerable to the effects of widespread damage associated with traumatic brain injury [37].

Results of HIT_SP_ for both playing levels collectively spanned from 10.7 to 207.5 with a median of 15.5. Differences between junior and senior players in the <21 and 21–63 values were negligible with 72% and 74% at the <21 and 23% at the 21–63 value, respectively. As these values represent approximately 95% of all impacts, it is clear that both levels are equal. However, junior players had significantly (*p* = <0.05) more impacts (35) at the >63 value than the seniors (17). Although these represented only 4% and 2% of impacts, respectively, a value of 63 is a 75% indicator for a concussive injury [32, 38] that can have significant implications on medical coverage for junior AF players. One area may be the game structure and speed that has changed significantly over the last 4 decades (Gray et al., 2010) with junior players still developing their skills through conditions to commensurate with their stage of learning and level of ability. Additionally, the junior players may be less disciplined in their play in comparison to the more experienced adult players.

### 4.2. Differences between Playing Positions

The results at various playing positions showed midfielders sustaining more impacts per player, per game over the course of the data collection period for junior and senior players. This study supports previous research [11], where it was also reported that AF midfielders experienced significantly more impacts per player. Additionally, the junior midfielders recorded significantly (*p* = 0.0443) higher median peak linear acceleration (15.5*g*) than defenders with the senior midfielders having significantly higher (*p* = 0.0221) median peak linear acceleration than forwards. Overall, senior midfielders recorded a higher median resultant peak linear acceleration (16.9*g*) than junior midfielders (15.5*g*; *p* = 0.0421). There is no data reporting which player position reports the highest level of SRC; however, why the midfield position in this study recorded more impacts needs to be examined in future research.

### 4.3. Association between Head Impacts and the Risk of Developing Sports-Related Concussion

This study included the RWE that allows identification of the variability of exposure from linear and rotational acceleration [17]. In order to accurately predict the risk of SRC, both linear and rotational acceleration should be accounted for to determine the concussion risk [17]. The RWE_CP_ enabled this concussion risk prediction to be undertaken. By recording the RWE_CP_ of individual players and player groups and for the sport [10], the resulting values may assist in identifying players with a potential cumulative exposure resulting in SRC. Previous studies in high school American football (14 to 18 yr.), Australian Football, under-11 junior rugby league, and collegiate wrestling [8, 11, 12, 17] have reported RWE_CP_. The RWE_CP_ values were evaluated by risk values of 25%, 25% to 75%, and >75% with the results showing similar mean values of 0.0008 for both junior and senior levels of participation. However, junior participants had a slightly higher percentage of impacts in the 0.2500–0.7500 (5.2 versus 3.9) and >0.7500 (3.3 versus 3.1) range than senior participants which again highlights the potential need for a change in current practice in AF that reflects potential increased risk of injury at a junior level. Player positions showed similar results between the three groups for the juniors but midfielders in the senior group recorded slightly higher RWE_CP_ values.

### 4.4. Informing Changes to Clinical Practice

Although RWE_CP_ were relatively small in both groups compared to American football (0.497) [17], AF (0.0003) [8] and junior rugby league (0.001) [10], the concern is the comparable level of the RWE_CP_ across junior and senior participants that places the juniors at a similar SRC risk as senior participants. Therefore, player safety at the junior level is one area that may be addressed with attention to SRC. The Australian Football League (AFL) has been proactive in SRC and first developed its community-AF concussion guidelines in 2011 based on the 2008 International Consensus Statement on Concussion in Sport [39]. The results of this study also indicate that current guidelines on appropriate medical cover for AF do not reflect the risks seen in previous research on SRC and this research on head impacts where senior games have higher levels of medical cover than junior levels.

Equipment and structural changes to the game at the junior level could be considered; however, current evidence does not support any protective effect for SRC from many of the soft-shell helmets available [40, 41]. Given the lack of effectiveness of headgear in protecting against SRC, other potential preventive solutions need to be measured for effectiveness. For example, future research could consider the effectiveness of training exercises for body-contact, neck strengthening, safe tackling, and reviewing the enforcement of games rules that all could be valuable strategies to reduce dangerous play that leads to injury [6]. Specifically, given the increased exposure seen in midfielders, targeting education strategies at this group may translate to decreases in the incidence of SRC. Our results support further inquiry as to when, why, and how SRCs occur to elucidate potential encompassing preventive measures.

### 4.5. Study Limitations

There are a several limitations in the study including data collection over a full season for each player and a small sample size. Furthermore, SRC assessments conducted by the team physiotherapist were not included in the analysis. The XPatch has undergone some reported validation studies and been compared with the Head Impact Telemetry System (HITS); however the results have varied [22, 42]. As a result of these different findings, the impact variables reported in this study should be assumed to have some form of error that is dependent on impact conditions and the measure of interest and the variability tested [29, 43]. There are no consistent reliability studies for the XPatch and the resultant impact variables that are recorded and the results presented in this study should be interpreted with some caution.

## 5. Conclusions

Obtaining head impact mechanics with the use of accelerometer sensors allows comparison between various levels of play and positions in AF. This is vital as those in the younger levels, whose brains are still developing, are potentially more susceptible to injury. In this study, the players at the junior level recorded similar levels of impact to those in the adult level with midfielders suffering the most impacts per match. Due to this, future research needs to address safety issues at the lower levels to ensure less traumatic impacts to the younger players and reviews of current practice in medical coverage for AF should be considered.

## Figures and Tables

**Table 1:** Impacts to the head greater than 10*g* for total impacts recorded, impacts by total, junior, and senior level teams and by forwards midfielders and defenders for each level of participation in an amateur Australian Football League team over a season of matches. Data are presented as median [interquartile range] and 95th percentile for total impacts, impacts per player position group, impact duration (ms), resultant linear and rotational acceleration, head impact telemetry severity profile, and risk weighted exposure combined probability.

	Total impacts *n* =	Per player per match season	Per player per match	Impact duration (ms)	PLA (*g*)		PRA (rad/s^2^)		HIT_SP_		RWE_CP_	
		Mean ± SD	Mean ± SD	Mean ± SD	Median [IQR]	95%	Median [IQR]	95%	Median [IQR]	95%	Median [IQR]	95%
Total												
Combined	1,609	60 ± 36	8 ± 8	10.9 ± 7.9	15.3 [11.9–23.3]	45.8	2,729.8 [1,680.0–4,784.3]	9,592.9	15.5 [13.2–20.8]	41.7	0.0008 [0.0003–0.0060]	0.9874
Junior	861	62 ± 39	7 ± 9	10.4^e^ ± 1.6	15.1 [11.9–22.2]	45.7	2,741.7 [1,712.8–4,648.4]	9,644.5	15.3 [12.6–21.2]	46.8	0.0008 [0.0003–0.0052]	0.3969
Senior	748	58 ± 35	8 ± 7	11.4^d^ ± 8.2	15.7 [11.9–24.2]	46.8	2,757.4 [1,652.6–4,915.7]	9,556.1	15.4 [13.0–21.2]	39.0	0.0008 [0.0003–0.0067]	0.3862

Junior												
Forwards	228^bc^	57 ± 17	7 ± 6	10.4^c^ ± 7.6	14.5^b^ [11.7–21.6]	40.5	2,493.1 [1,511.3–4,760.5]	9,427.4	15.4 [11.7–26.1]	98.0	0.0006 [0.0002–0.0061]	0.3484
Midfielder	356^ac^	71 ± 60	8 ± 12	11.0^cd^ ± 8.1	15.5^ace^ [12.0–22.5]	47.3	2,676.3 [1,718.5–4,808.0]	10,672.9	15.2 [12.7–20.4]	46.8	0.0007 [0.0003–0.0063]	0.6233
Defender	277^ab^	55 ± 30	8 ± 5	9.6^abd^ ± 6.3	14.8^be^ [11.9–22.2]	39.5	2,815.1 [1,848.2–4,433.0]	8,406.2	15.4^e^ [12.9–21.6]	42.7	0.0008 [0.0003–0.0040]	0.2035

Senior												
Forwards	236^b^	59 ± 27	9 ± 7	10.0^b^ ± 7.0	15.8^b^ [11.7–24.3]	45.2	2,701.0 [1,623.5–5,235.5]	9,766.7	14.2 [11.6–22.1]	46.0	0.0008 [0.0003–0.0101]	0.4802
Midfielder	305^ac^	76 ± 54	11 ± 11	13.1^ae^ ± 9.6	16.9^ad^ [12.5–26.5]	52.8	2,963.1 [1,744.6–5,068.2]	9,617.2	15.1 [13.1–21.2]	43.1	0.0010 [0.0003–0.0082]	0.4660
Defender	207^b^	41 ± 12	8 ± 7	10.9^e^ ± 7.2	14.7^d^ [11.9–21.4]	43.1	2,519.2 [1,569.7–4,560.7]	8,599.6	15.8^d^ [13.6–20.6]	37.5	0.0006 [0.0003–0.0042]	0.2701

[IQR] = interquartile (25th to 75th) percentile; 95% = 95th percentile; PLA (*g*) = peak linear acceleration in gravitational force (*g*); PRA (rad/s^2^) = peak rotational acceleration in radians/second^2^; HIT_SP_ = Head Impact Telemetry Severity Profile; RWE_CP_ = Risk Weighted Exposure Combined Probability; significant difference (*p* < 0.05) compared to ^a^forward; ^b^midfielder; ^c^defender; ^d^junior; ^e^senior.

**Table 2:** Impacts to the head greater than 10*g* by impact location for total impacts, impacts recorded by junior and senior players in an amateur Australian Football League team over a season of matches.

Location	*n* =	%	Duration ms ± SD	PLA (*g*)		PRA (rad/s^2^)		HIT_SP_		RWE_CP_	
				Median [IQR]	0.95	Median [IQR]	0.95	Median [IQR]	0.95	Median [IQR]	0.95
Total											
Front	563^df^	35.0	11.0 ± 7.6	16.2 [12.2–24.6]	44.8	3,278.8^ef^ [2,050.0–5,212.4]	9,684.3	15.6^f^ [13.0–21.4]	39.2	0.0012^ef^ [0.0004–0.0085]	0.5046
Back	368^cef^	22.9	10.2 ± 7.6^e^	15.5 [11.7–23.5]	42.4	2,705.7 [1,343.1–4,873.5]	8,832.4	14.6 [11.5–23.0]	46.0	0.0007 [0.0002–0.0065]	0.2269
Side	602^df^	37.4	11.1 ± 8.1^d^	14.9 [11.8–21.9]	51.3	2,389.9^c^ [1,517.6–4,294.6]	9,728.9	15.4 [13.6–20.2]	42.3	0.0006^c^ [0.0002–0.0037]	0.4449
Top	76^cef^	4.7	13.0 ± 10.2	16.0 [11.8–22.1]	48.2	2,310.3^c^ [1,469.0–4,458.2]	11,139.3	14.6^c^ [12.5–19.4]	53.6	0.0005^c^ [0.0002–0.0042]	0.7728

Juniors											
Front	326^bdef^	37.9	10.8 ± 7.3	15.8 [12.1–23.9]	44.0	3,115.8^de^ [2,062.2–5,213.4]	9,640.6	14.6 [11.4–20.7]	48.0	0.0010^de^ [0.0004–0.0079]	0.5156
Back	215^cef^	25.0	10.2 ± 7.8	15.5 [11.8–22.9]	40.3	2,705.7^c^ [1,348.7–4,653.7]	8,839.1	14.5 [12.0–23.2]	47.1	0.0007^c^ [0.0002–0.0047]	0.2247
Side	277^bcdf^	32.2	10.0 ± 7.1	14.2 [11.8–20.5]	49.1	2,450.2^c^ [1,527.0–3,944.1]	10,267.7	15.4 [13.5–19.9]	45.1	0.0006^c^ [0.0002–0.0027]	0.5151
Top	43^cde^	5.0	11.4 ± 9.9	16.1 [11.7–21.1]	44.0	2,302.9 [1,562.9–3,823.5]	10,491.6	15.6 [12.9–19.2]	47.9	0.0005 [0.0003–0.0021]	0.5334

Seniors											
Front	237^adef^	31.7	11.2 ± 8.0^f^	15.2 [11.7–22.8]	46.0	2,334.9 [1,481.9–4,363.1]	10,158.3	15.3 [13.4–20.4]	35.6	0.0005 [0.0002–0.0039]	0.5140
Back	153^cef^	20.5	10.2 ± 7.2^f^	15.3 [11.7–24.6]	43.3	2,711.6 [1,302.2–5,187.3]	8,841.5	14.2 [10.5–23.4]	46.3	0.0008 [0.0002–0.0080]	0.2271
Side	325^acdf^	43.4	12.0 ± 8.7	14.6 [11.6–20.7]	53.2	2,198.3 [1,441.5–4,061.1]	9,028.1	14.9 [13.3–18.8]	40.4	0.0005 [0.0002–0.0029]	0.3909
Top	33^cde^	4.4	14.8 ± 10.3^ce^	16.0 [12.2–32.8]	58.0	2,760.9 [1,110.3–4,916.3]	12,029.2	13.5 [11.8–23.9]	67.8	0.0008 [0.0002–0.0105]	0.9019

Data are presented as mean (±SD) for impact duration (ms) and median [IQR] and 95th percentile for total impacts, juniors, seniors, resultant linear, and rotational acceleration, Head Impact Telemetry Severity Profile, and Risk Weighted Exposure Combined Probability. IQR: = interquartile (25th to 75th) percentile; 95%: = 95th percentile; PLA (*g*): peak linear acceleration in gravitational force (*g*); PRA (rad/s^2^): = peak rotational acceleration in radians per second; HIT_SP_: = Head Impact Telemetry Severity Profile; RWE_CP_: = Risk Weighted Exposure Combined Probability; significant difference (*p* < 0.05) compared to ^a^junior; ^b^senior; ^c^front; ^d^back; ^e^side; ^f^top.

**Table 3:** Impacts to the head greater than 10*g* by injury tolerance, injury severity for resultant linear and rotational accelerations, Head Impact Telemetry Severity Profile and Risk Weighted Exposure Combined Probability for total impacts, impacts recorded by junior, senior, and total in an amateur Australian Football League team over a season of matches.

	Junior	Senior	Total
	*n* = (%)	*n* = (%)	*n* = (%)
Injury tolerance			
>95*g*	2 (0.2)	6 (0.8)	8 (0.5)
>5500 rad/s^2^	153 (17.8)	148 (19.8)	301 (18.7)

Injury severity (linear)			
<66*g*	821 (95.4)	723 (96.7)	1,544 (96.0)
66–106*g*	31 (3.6)	18 (2.4)	49 (3.0)
>106*g*	9 (1.0)	7 (0.9)	16 (1.0)

Injury severity (rotational)			
<4600 rad/s^2^	630 (73.2)	531 (71.0)	1,161 (72.2)
4600–7900 rad/s^2^	155 (18.0)	150 (20.1)	305 (19.0)
>7900 rad/s^2^	76 (8.8)	67 (9.0)	143 (8.9)

HIT_SP_			
<21	627 (72.8)	554 (74.1)	1,181 (73.4)
21–63	199 (23.1)	177 (23.7)	376 (23.4)
>63	35^b^ (4.1)	17^a^ (2.3)	52 (3.2)

RWE_CP_			
<0.2500	788 (91.5)	696 (93.0)	1,484 (92.2)
0.2500–0.7500	45 (5.2)	29 (3.9)	74 (4.6)
>0.7500	28 (3.3)	23 (3.1)	51 (3.2)

Data are presented as number of impacts and percentage of impacts recorded. rad/s^2^: radians per second; HIT_SP_: Head Impact Telemetry Severity Profile; RWE_CP_: Risk Weighted Exposure Combined Probability; significant difference (*p* < 0.05) compared ^a^junior; ^b^senior.
